# The Sonozotz project: Assembling an echolocation call library for bats in a megadiverse country

**DOI:** 10.1002/ece3.6245

**Published:** 2020-05-18

**Authors:** Veronica Zamora‐Gutierrez, Jorge Ortega, Rafael Avila‐Flores, Pedro Adrián Aguilar‐Rodríguez, Martín Alarcón‐Montano, Luis Gerardo Avila‐Torresagatón, Jorge Ayala‐Berdón, Beatriz Bolívar‐Cimé, Miguel Briones‐Salas, Martha Chan‐Noh, Manuel Chávez‐Cauich, Cuauhtémoc Chávez, Patricia Cortés‐Calva, Juan Cruzado, Jesús Carlo Cuevas, Melina Del Real‐Monroy, Cynthia Elizalde‐Arellano, Margarita García‐Luis, Rodrigo García‐Morales, José Antonio Guerrero, Aldo A. Guevara‐Carrizales, Edgar G. Gutiérrez, Luis Arturo Hernández‐Mijangos, Martha Pilar Ibarra‐López, Luis Ignacio Iñiguez‐Dávalos, Rafael León‐Madrazo, Celia López‐González, M. Concepción López‐Téllez, Juan Carlos López‐Vidal, Santiago Martínez‐Balvanera, Fernando Montiel‐Reyes, Rene Murrieta‐Galindo, Carmen Lorena Orozco‐Lugo, Juan M. Pech‐Canché, Lucio Pérez‐Pérez, María Magdalena Ramírez‐Martínez, Areli Rizo‐Aguilar, Everardo Robredo‐Esquivelzeta, Alba Z. Rodas‐Martínez, Marcial Alejandro Rojo‐Cruz, Celia Isela Selem‐Salas, Elena Uribe‐Bencomo, Jorge A. Vargas‐Contreras, M. Cristina MacSwiney G.

**Affiliations:** ^1^ CONACYT—Centro Interdisciplinario de Investigación para el Desarrollo Integral Regional Unidad Durango (CIIDIR) Instituto Politécnico Nacional Durango México; ^2^ Departamento de Zoología Escuela Nacional de Ciencias Biológicas Instituto Politécnico Nacional Ciudad de México México; ^3^ División Académica de Ciencias Biológicas Universidad Juárez Autónoma de Tabasco Villahermosa México; ^4^ Centro de Investigaciones Tropicales Universidad Veracruzana Xalapa México; ^5^ Universidad Autónoma de Tlaxcala Tlaxcala de Xicohténcatl México; ^6^ Facultad de Ciencias Biológicas Universidad Autónoma del Estado de Morelos Cuernavaca México; ^7^ CONACYT Universidad Autónoma de Tlaxcala Tlaxcala de Xicohténcatl México; ^8^ Instituto de Investigaciones Forestales Universidad Veracruzana Xalapa México; ^9^ Centro Interdisciplinario de Investigación para el Desarrollo Integral Regional Unidad Oaxaca (CIIDIR) Instituto Politécnico Nacional Oaxaca México; ^10^ Campus de Ciencias Biológicas‐Agropecuarias Universidad Autónoma de Yucatán Mérida México; ^11^ Departamento de Ciencias Ambientales Universidad Autónoma Metropolitana‐Unidad Lerma Lerma México; ^12^ Programa de Planeación Ambiental y Conservación Centro de Investigaciones Biológicas del Noroeste, S.C. La Paz México; ^13^ Independent Biologist Mérida México; ^14^ Ingeniería en Recursos Naturales y Agropecuarios Universidad de Guadalajara Autlán México; ^15^ Laboratorio de Genómica Evolutiva Universidad Autónoma de Zacatecas Zacatecas México; ^16^ Instituto Tecnológico del Valle de Oaxaca Xoxocotlán México; ^17^ Centro del Cambio Global y la Sustentabilidad Villahermosa México; ^18^ Facultad de Ciencias Universidad Autónoma de Baja California Ensenada México; ^19^ Tierra Verde Naturaleza y Cultura Tuxtla Gutiérrez México; ^20^ Departamento de Ecología y Recursos Naturales Universidad de Guadalajara Autlán México; ^21^ Centro Interdisciplinario de Investigación para el Desarrollo Integral Regional Unidad Durango (CIIDIR) Instituto Politécnico Nacional Durango México; ^22^ Facultad de Ciencias Biológicas Benemérita Universidad Autónoma de Puebla Puebla México; ^23^ Comisión Nacional para el Conocimiento y Uso de la Biodiversidad Ciudad de México México; ^24^ Desarrollo Regional Sustentable El Colegio de Veracruz Xalapa México; ^25^ Centro de Investigación en Biodiversidad y Conservación Universidad Autónoma del Estado de Morelos Cuernavaca México; ^26^ Facultad de Ciencias Biológicas y Agropecuarias Universidad Veracruzana Tuxpan México; ^27^ Departamento de Ciencias de la Salud y Ecología Humana Universidad de Guadalajara Autlán México; ^28^ Facultad de Ciencias Químicas e Ingeniería Universidad Autónoma del Estado de Morelos Cuernavaca México; ^29^ Facultad de Ciencias Químico Biológicas Universidad Autónoma de Campeche Campeche México

**Keywords:** acoustics, Chiroptera, insectivorous bats, Neotropics, ultrasounds

## Abstract

Bat acoustic libraries are important tools that assemble echolocation calls to allow the comparison and discrimination to confirm species identifications. The Sonozotz project represents the first nation‐wide library of bat echolocation calls for a megadiverse country. It was assembled following a standardized recording protocol that aimed to cover different recording habitats, recording techniques, and call variation inherent to individuals. The Sonozotz project included 69 species of echolocating bats, a high species richness that represents 50% of bat species found in the country. We include recommendations on how the database can be used and how the sampling methods can be potentially replicated in countries with similar environmental and geographic conditions. To our knowledge, this represents the most exhaustive effort to date to document and compile the diversity of bat echolocation calls for a megadiverse country. This database will be useful to address a range of ecological questions including the effects of anthropogenic activities on bat communities through the analysis of bat sound.

## Graphical abstract

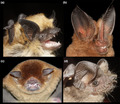

## 1 INTRODUCTION

**FIGURE 1:**
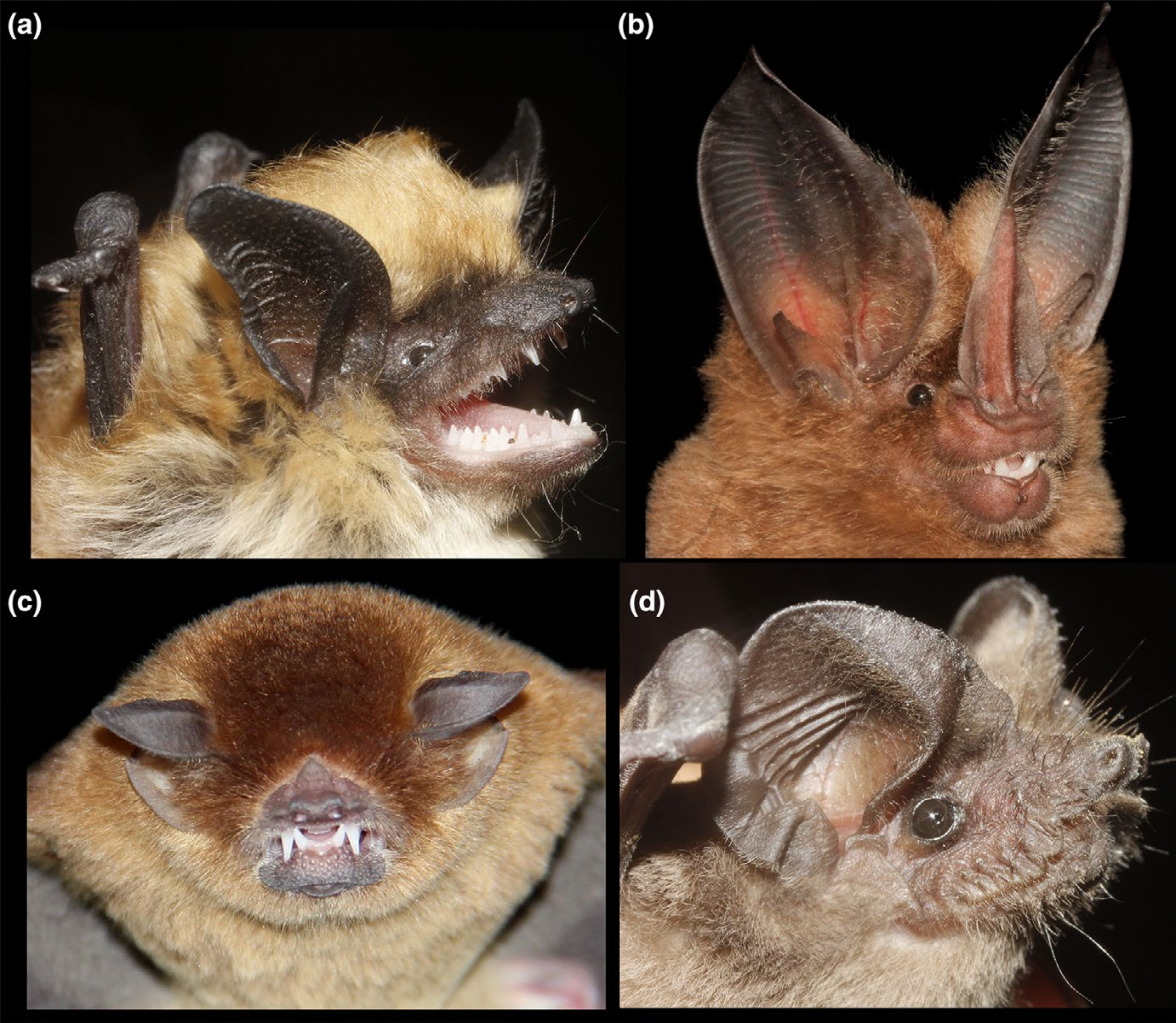
Mexican bats representative of different echolocation strategies. (a) *Myotis californicus* belongs to the Vespertilionidae family whose echolocation calls can be characterized by presenting broadband‐modulated frequencies, which are of relatively high intensity and short duration; (b) *Mimon cozumelae* is a member of the Phyllostomidae family and emits echolocation calls (usually of low intensity) composed of multiharmonic components and constant modulated frequencies; (c) *Pteronotus parnellii* emits typical calls of the Mormoopidae family consisting of a constant frequency segment, followed by a modulated sweep descendent call, and finalizing with a quasi‐constant frequency with a short duration, and it is the only species in America that compensates for the Doppler effect; (d) *Tadarida brasiliensis* is a representative of the Molossidae family with typical echolocation calls of open space foragers with relatively low frequencies and long call durations

Bats are a unique group of mammals characterized by their ability to fly and to echolocate. Both characteristics have allowed bats to adapt to a large number of environments and to have broad geographic distributions. Echolocation is a mechanism used by bats for orientation and food detection, in which they emit ultrasonic pulses that bounce and return to the source in the form of echoes (Liu et al., 2010). Bats have adapted to a large number of feeding habits that range from generalist insectivorous to specialized frugivorous with a marked correlation between their feeding preferences and their echolocation characteristics (Figure 1; Denzinger & Schnitzler, 2013). For example, insectivorous bats that forage in open spaces use long and low‐frequency pulses to detect distant objects and prey. In contrast, bat species adapted to forage in cluttered environments emit short, broadband, high‐frequency pulses that bounce off potential objects and prey at close distances, giving the animal a very precise spatial configuration scheme (Fenton, Portfors, Rautenbach, & Waterman, 1998).

Bats occupy a wide diversity of niches which allows them to perform a variety of ecosystem roles, including pollination, seed dispersal, and suppression of insects (Jones, Jacobs, Kunz, & Racey, 2009; Park, 2015; Russo & Jones, 2015; Stahlschmidt & Brühl, 2012). These unique characteristics, in addition to their longevity, low reproductive rate and mobility, have made them ideal indicators for monitoring programs to help us better understand the effects of global anthropogenic change on biodiversity (Jones et al., 2009). Over the last decades, ultrasonic bat detectors equipped with highly sensitive microphones have allowed the recording of echolocation pulse emissions of diverse frequencies and intensities. At present, bat detectors have become the most popular tools to characterize bat faunas at local, regional, or even broader scales (Armitage & Ober, 2010; Blumstein et al.., 2011; Clement, Murray, Solick, & Gruver, 2014; Jones et al., 2009).

Accurate data on species composition based on ultrasound detection require the use of standardized methods. Systematic bat bioacoustics surveys and monitoring programs can reveal indications of environmental changes, ecological disturbances, and anthropogenic habitat modification, as they provide information on activity patterns of different species. Acoustic detection, in combination with complementary methodologies (e.g., morphometry and molecular biology), can be an excellent tool to have a precise characterization of bat assemblages (Frick, 2013; Meyer, 2015). Specifically, acoustic identification of bats is a noninvasive technique that provides accurate information regarding the presence and activity of most high‐intensity echolocating species at relatively low costs (Clement et al., 2014). However, acoustic methods should be used considering its limitations and biases. Likelihood of detection varies, as with most survey methods, depending on the species echolocation type, habitat, and environmental conditions (Hayes, 2000; Jones & Teeling, 2006; Meyer et al., 2011; Patriquin, Hogberg, Chruszcz, & Barclay, 2003). Another difficulty with acoustic methods is to determine abundances since it is difficult to differentiate between recordings from single or multiple individual of the same species (Hayes, Ober, & Sherwin, 2009).

Recordings from free‐flying bats must be compared with reliable field acoustic identifications in order to classify the unknown calls to different taxonomic and ecological levels (Russo & Voigt, 2016; Zamora‐Gutierrez et al., 2016). Classification of unknown calls has been traditionally done by comparing published data in the literature that come from different geographical regions, under environmental and/or methodological conditions that are not necessarily comparable (Braun de Torrez, Wallrichs, Ober, & McCleery, 2017). A collection of bat calls recorded within the variety of Mexican ecosystems is needed as a standard reference for regional bat acoustic studies. In the past two decades, computational algorithms have allowed discrimination between some species in free‐flying bat recordings. Automated acoustic analysis can greatly diminish the amount of human labor in bat studies and is a vital tool for large‐scale acoustic monitoring systems (Armitage & Ober, 2010). Yet, increasing the accuracy of automated detection and classification methods requires the collection of sufficient field data that captures regional particularities of bat acoustic phenotypes for each target group.

The identification of bat acoustic signals among the environmental noise is facilitated because bat calls have very distinctive patterns over a range of frequencies (>9 kHz) that very few taxa use (Walters et al., 2013). However, identification of bat calls to species level is challenging because (a) sensory and ecological constraints, monophyly, as well as phylogenetic convergence, have led to overlapping characteristics between some bat groups (e.g., *Myotis*), complicating the acoustic identification of some species (Jones & Holderied, 2007; Jones & Teeling, 2006); (b) bat acoustic signals are highly diverse and have high levels of plasticity, presenting a great amount of intra‐ and interspecific variation, and (c) because of the high overlap between species of some groups (Jung, Molinari, & Kalko, 2014; Kalko & Schnitzler, 1993; Murray, Britzke, & Robbins, 2001; Thomas, Bell, & Fenton, 1987). In addition to the complex variation of bat calls and the high similarity between some species, echolocation calls of many species are not well documented, especially from tropical and megadiverse regions (Walters et al., 2013; Zamora‐Gutierrez et al., 2016).

Call libraries are an important resource for assembling, accessing, and interpreting echolocation call characteristics of bats and provide comparative data for species acoustic identifications (Szewczak, 2002). Bat acoustic libraries must have a good documentation of as much variation of bat calls of the same species as possible to adequately characterize bat acoustic diversity (Walters et al., 2013). Call structure between individuals of the same species can vary based upon anatomical differences associated with geographic location, sex, and age of the individual (Jung et al., 2014; Kalko & Schnitzler, 1993; Murray et al., 2001; Obrist, 1995; Siemers, Beedholm, Dietz, Dietz, & Ivanova, 2005; Thomas et al., 1987). Intraspecific variation of bat calls can also depend on adjustments made by individuals in response to environmental conditions, foraging tactics, and in the presence of conspecifics (Kalko & Schnitzler, 1993; Obrist, 1995; Ulanovsky, Fenton, Tsoar, & Korine, 2004). However, variability in bat call structure can be generated by artifacts resulting from recording methods (Barclay, 1999; Obrist, 1995; Szewczak, 2002), including the release of individuals under unusual environmental conditions (e.g., vegetation with structural characteristics distinct to those encountered in the foraging grounds of the species; Patriquin et al., 2003) and recording techniques (i.e., zip‐lining and hand release).

Here, we present and describe the first nation‐wide library of bat echolocation calls for a megadiverse country. Our project, Sonozotz‐AMMAC‐CONABIO (hereafter referred as Sonozotz), was assembled following a standardized recording protocol to reduce variation on bat calls resulting from unrecorded technical issues and to avoid biases toward specific habitats and recording methods. For most bats, we included a set of different recording habitats (e.g., close vegetation, open environments, over water), recording techniques (e.g., in hand, hand release, zip‐lining, take‐off flight from perch), and call variation inherent to individuals (i.e., sex, age, geographic location). The Sonozotz project included 69 species of echolocating bats, a high species richness that represents 50% of bat species found in the country. We include recommendations on how the database can be used and how the sampling methods can be potentially replicated in other countries.

## 2 MATERIALS AND METHODS

### 2.1 Geographic and environmental coverage

**FIGURE 2:**
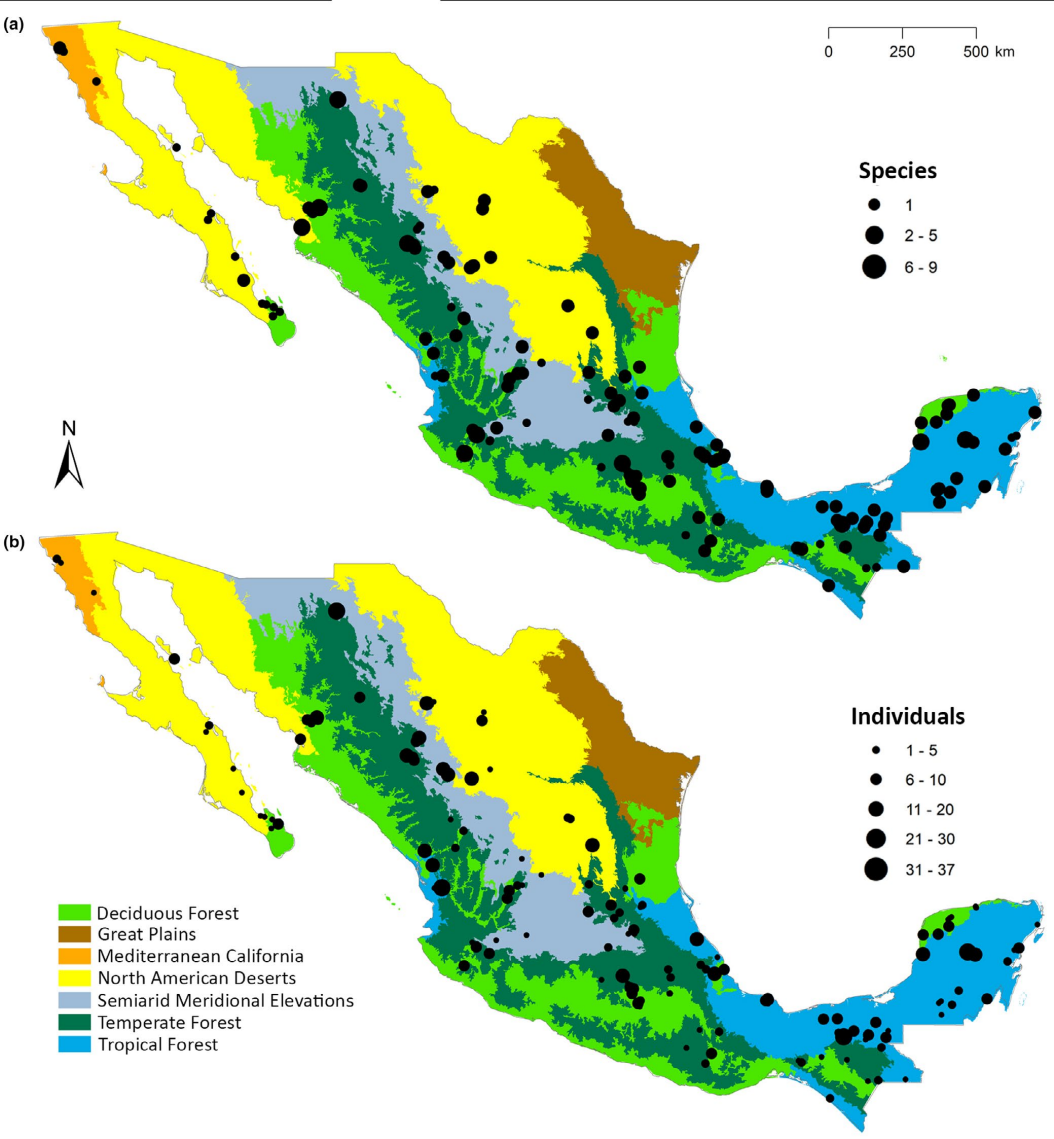
Sampling localities of the project in each of the seven ecoregions of Mexico defined by CONABIO (INEGI, CONABIO & INE, 2008): (a) number of species sampled in each locality, and (b) number of individuals recorded in each locality

We selected multiple localities scattered across Mexico to maximize the number of species included in the database. We divided the Mexican territory into eight study regions based on topography, environmental complexity, and the collaboration of bat experts working in each region: (a) Californian region (Baja California, Baja California Sur, and Sonora); (b) Northeast region (Durango, Sinaloa, and Chihuahua); (c) West region (Colima, Nayarit, and Jalisco); (d) East region (Puebla, Tlaxcala, and Veracruz): (e) North center region (Aguascalientes, Guanajuato, San Luis Potosí, Nuevo León, and Zacatecas); (f) South center region (Estado de México, Morelos, Hidalgo, and Querétaro); (g) Southeast region (Campeche, Quintana Roo, and Yucatán); and (h) Southwest region (Chiapas, Oaxaca, and Tabasco). Number of individuals recorded per species in each region is presented in Table 1. Based on this organization, we sampled in 27/32 (84%) of the Mexican states and six out of the seven ecoregions (defined as geographically distinctive areas containing a group of natural communities that share most of their species, environmental conditions, and ecological dynamics; Challenger & Soberón, 2008) that have been defined for the Mexican territory by the National Commission for the Knowledge and Use of Biodiversity (CONABIO; Figure 2, INEGI, CONABIO, & INE, 2008). The localities sampled covered an altitudinal gradient ranging from sea level to 3,600 m.a.s.l., and a great variety of ecosystems ranging from the northern xerophytic shrublands to the southeastern tropical forests. Due to logistics and security reasons, the states of Tamaulipas, Michoacán, Guerrero, and Ciudad de México, as well as the Great Plains (GP) ecoregion, were not sampled.

**TABLE 1:** Total number individuals recorded for species in each region. R1 = Region 1… R8 = Region 8

Family/Especie	R1	R2	R3	R4	R5	R6	R7	R8	Total
Emballonuridae									
*Balantiopteryx io*								9	9
*Balantiopteryx plicata*		2	11	5	10	9		6	43
*Peropteryx kappleri*								5	5
*Peropteryx macrotis*							7	15	22
*Rhynchonycteris naso*							6	16	22
*Saccopteryx bilineata*				9			12	9	30
Molossidae									
*Eumops nanus*							1		1
*Eumops perotis*		1							1
*Molossus alvarezi*							5		5
*Molossus molossus*			1					2	3
*Molossus rufus*				14	7		22	11	54
*Molossus sinaloae*						5			5
*Nyctinomops aurispinosus*	3								3
*Nyctinomops femorosaccus*					2				2
*Nyctinomops laticaudatus*							10		10
*Nyctinomops macrotis*						2			2
*Tadarida brasiliensis*	26	75	7		17	16		13	154
Mormoopidae									
*Mormoops megalophylla*		36	4	11	16	10	20	18	115
*Pteronotus davyi*		17	1	12	3		23	35	91
*Pteronotus gymnonotus*								7	7
*Pteronotus parnellii*	9	9	15	25	18	26	43	19	164
*Pteronotus personatus*		12		12	5		7	4	40
Natalidae									
*Natalus mexicanus*		13	2	3		10	8		36
Noctilionidae									
*Noctilio leporinus*							9		9
Phyllostomidae									
*Chrotopterus auritus*							4	3	7
*Lampronycteris brachyotis*							1		1
*Lonchorhina aurita*							1		1
*Lophostoma brasiliense*				1					1
*Macrotus californicus*	25	18							43
*Macrotus waterhousii*				2		2			4
*Micronycteris microtis*				1	2	1	2	1	7
*Mimon cozumelae*							7	4	11
*Phyllostomus discolor*								1	1
*Trachops cirrhosus*							4	1	5
*Vampyrum spectrum*								1	1
Vespertilionidae									
*Antrozous pallidus*	1	52				1			54
*Bauerus dubiaquercus*		1							1
*Corynorhinus mexicanus*			2	6					8
*Corynorhinus townsendii*		5			14	10		1	30
*Eptesicus brasiliensis*					1			2	3
*Eptesicus furinalis*			1				9	6	16
*Eptesicus fuscus*	14	27	3	3	4	4		3	58
*Idionycteris phyllotis*								1	1
*Lasiurus blossevillii*	3	3	4	2		2		2	16
*Lasiurus cinereus*	2	7		4	1	10		1	25
*Lasiurus ega*				1	2		5	1	9
*Lasiurus intermedius*			2			2			4
*Lasiurus xanthinus*	3	1	1						5
*Myotis albescens*				5					5
*Myotis auriculus*	1	2			3				6
*Myotis californicus*	2	5	1	6	20	7			41
*Myotis elegans*				2				6	8
*Myotis fortidens*			1					4	5
*Myotis keaysi*				5	2	11	11	4	33
*Myotis melanorhinus*		10		1	5				16
*Myotis nigricans*			1	27		5		21	54
*Myotis occultus*			1						1
*Myotis peninsularis*	12								12
*Myotis thysanodes*		2	1	2					5
*Myotis velifer*	7	124	1	9	23	14		10	188
*Myotis vivesi*	19								19
*Myotis volans*	9	3				2			14
*Myotis yumanensis*	1	36			11	8			56
*Parastrellus hesperus*	2	7				3			12
*Perimyotis subflavus*					1				1
*Rhogeessa aeneus*							11		11
*Rhogeessa alleni*				1		1			2
*Rhogeessa parvula*	11		2	1		2			16
*Rhogeessa tumida*				7	1	2		4	14
Total number of individuals	150	468	62	177	168	165	228	246	1,664

Sampling efforts in each study region were coordinated by experienced bat researchers belonging to local and federal universities, NGO's, and governmental agencies and were carried out between June 2016 and December 2017. In each study region, we selected at least eight sites of biological interest or conservation priority (e.g., high species richness, presence of endemic species, and specific roosting sites), always intending to maximize the geographic coverage and the inclusion of a wide variety of habitats and environmental conditions. Sites included relatively large areas such as biosphere reserves, national parks, basins, valleys, urban landscapes, or geopolitical entities (e.g., ejidos, municipalities). In each site, at least one sampling point or locality was established. Each locality could include a single point in space defined by a unique geographic coordinate (e.g., cave entrance, small pond, single building), or a small area where several capture points in proximity to each other were established (e.g. small forest fragment, lake, urban park, a portion of a biosphere reserve). Sampling season was selected based on the climatic characteristics of each sampling region. For example, in several northern localities, sampling was avoided during the winter season, considering the possibility that many local bat populations either hibernate or migrate.

To exemplify the spatial coverage achieved in this database, we produced potential distribution maps showing sampling localities within this study for three insectivorous species: (a) *Tadarida brasiliensis*, a member of the Molossidae family with a wide distribution all over Mexico; (b) *Myotis yumanensis*, a vespertilionid species with Nearctic affinity; and (c) *Pteronotus davyi*, a species with Neotropical affinity belonging to the Mormoopidae family. The suitability models and occurrence records to delineate the distribution maps were obtained from Zamora‐Gutierrez, Pearson, Green, and Jones (2018). We used these distributional points to construct a distributional range for each species by means of an alpha hull polygon using a buffer of 20 km around occurrence points. Distributional ranges for each species were transformed into a “SpatialPolygonDataFrame” and recorded as shape files. These shape files were used to limit the projections of the suitability models to the potential distribution areas of each species (Figure 4). This analysis was done in R program v. 3.5.2 (R Development Core Team, 2018).

### 2.2 Capture, handling, and sampling of bats

Specific techniques for capturing, handling, and sound‐recording bats were defined in collaborative workshops attended by groups of bat specialists, mathematicians, and computational scientists. The procedures that were standardized in this project included labeling of biological samples, photographs, and sound files, as well as definition of field datasheets and database formats. Attendance to any of the workshops and training to handle the recording equipment used to compile the database was a requisite to become a collector for the project. A total of 63 people were trained in a total of four workshops.

In each sampling locality, we placed mist‐nets of different sizes (6, 9, or 12‐m long) at points and positions that maximized capture probability such as flying paths, trails, streams, forest edges, tree lines, ponds, and roosts (caves, buildings, bridges, crevices, or tree holes). There were few instances, especially at cave entrances and small cave chambers, in which harp traps and hand nets were preferred for mist‐nets to reduce stress over the animals and avoid injuring them. Captured animals were identified to species level following specialized taxonomic keys (Álvarez‐Castañeda, Álvarez, & González‐Ruiz, 2015; Medellín, Arita, & Sánchez, 2008; Reid, 2009). Scientific names were standardized according to Ramírez‐Pulido, González‐Ruiz, Gardner, and Arroyo‐Cabrales (2014), but we considered that Natalidae contains only one species, *Natalus mexicanus* (López‐Wilchis et al., 2012), and that *Molossus sinaloae* and *M. alvarezi* are different species (González‐Ruiz, Ramírez‐Pulido, & Arroyo‐Cabrales, 2011). For this study, we also include the echolocation calls of species from the Glyphonycterinae, Macrotinae, Micronycterinae, and Phyllostominae subfamilies (Phyllostomidae), as most of these species base their diet in insects and also because some species have proven to emit intense and distinct vocalizations that resemble those of aerial insectivorous and trawling bats of other families (Gessinger, Gonzalez‐Terrazas, Page, Jung, & Tschapka, 2019; Weinbeer, Kalko, & Jung, 2013).

For each individual, we recorded sex, age (juvenile or adult), reproductive status (females: inactive, pregnant, or lactating; males: abdominal, inguinal or scrotal testes), and standard morphometric measurements (forearm length, head and body length, tail length, and body weight). Individual bats were photographed in standard formats and angles to provide support for posterior taxonomic identification and to create a photographic library of Mexican insectivorous bats. In addition, we obtained a small wing biopsy (diameter = 2 mm) stored in 96% ethanol to also serve as genetic reference material for future studies, in the case that intraspecific acoustic variation can hint to the presence of cryptic species.

When identification certainty was <80% (based on the judgment of the most experienced collector), a voucher specimen was collected to confirm its identity based on cranial and postcranial characters and measurements. All handling and sampling procedures followed ethical recommendations provided by Sikes and the Animal Care and Use Committee of the American Society of Mammalogists (2016). This project had collection permits (SGPA/DGVS/05867/16, SGPA/DGVS/07291/17) issued by the Secretaría de Medio Ambiente y Recursos Naturales to M. Briones‐Salas.

Individual data and biopsy samples were labeled with unique consecutive numbers which kept information on the region, site, and locality and will be freely available to researchers together with the acoustic material. Collected tissues were deposited in the Regional Collection of Durango (Mammalia), at CIDIIR‐Durango, and voucher specimens were deposited at the Mammalogy Collection, CIB at Centro de Investigaciones Biológicas del Noroeste (CIBNOR).

### 2.3 Recording of bat echolocation calls

The ultimate purpose of the Sonozotz project was to build a reference call library that could be used to identify free‐flying bats while foraging or commuting under natural conditions. Therefore, we aimed to record search calls (defined as species‐specific signal types that are intimately linked to the ecological conditions encountered by bats that are navigating and/or searching for food‐ Schnitzler, Moss, & Denzinger, 2003) under the conditions most commonly encountered by the species depending on their traits, habits, and behavior. We used literature information and personal experience to classify bat species on six recording categories, based on flight and echolocation attributes, and defined the corresponding settings and conditions of recording for each group (Brigham, Kalko, Jones, Parsons, & Limpens, 2004; Denzinger & Schnitzler, 2013; Kalko, Handley, & Handley, 1996; Mora & Torres, 2008; Smotherman & Guillén‐Servent, 2008). Species‐specific recommendations included recording mode: (a) hand release at ground level: Bats were released from 1.5–2 m from the ground; (b) hand release at heights > 5 m; (c) flight cage: rooms or enclosures that allowed the bat to fly, we used this technique for species low‐intensity calls (e.g., *Lampronycteris brachyotis, Lophostoma brasiliensis*); (d) zip‐lining: bats are attached to a 2‐m length of small elastic cord by a loose‐fitting loop of the cord pulled over the bat's foot, the other end of the elastic cord is attached via a small snap swivel to 30–50 m of taut monofilament line about 1 m above the ground; (e) inside the bag; and (f) take‐off flight from perch, distance to microphone (0.5, 0.5–1, 5, or >10 m). We also described the recording environment (stationary inside bag, closed, edge, or open). Bat echolocation calls were recorded immediately after processing individuals, which were afterward released on site. All bats were recorded in real time with broadband bat detectors (Avisoft UltraSoundGate 116H; Avisoft Bioacoustics), coupled with a sensitive condenser microphone (CM16/CMPA; Avisoft Bioacoustics) through a XLR‐5 cable, and a laptop Dell Inspiron 7348 (Dell Inc.) running the software Avisoft‐RECORDER (Avisoft Bioacoustics) through a USB cable.

Recording settings were fixed on the software using standard parameters. We recorded in channel 1 without going over 15 s per recording sequence employing a sampling rate of 300 kHz, a sample resolution of 16 bits, and a high‐pass filtering of 4 kHz. We named files directly on the software window typing the unique codes previously assigned that preserved information on the region, site, locality, date, time, and individual. Complementary information was recorded on channel 2 (voice notes) to store information on environmental conditions, recording mode, and any other significant information for the recording output. Parameters were discussed and agreed in a workshop which attended general and regional coordinators. Sequences of 15 s were set in order to avoid heavy files; sampling rate of 300 kHz was selected as this rate covers all species frequencies of Mexican species; sample resolution was decided on the basis of previous recordings with the same microphone; a high‐pass filtering of 4 kHz was selected to reduce low‐frequency sounds. The species with lowest frequency in Mexico is *Eumops perotis* (ca. 7 kHz); therefore, using this filter did not reduce the possibility of recording the species. It is important to mention that all recording settings were checked with experts from Avisoft Company in order to obtain the best recordings from the recorder (R. Specht, personal communication)

### 2.4 Sound analyses

We used BATSOUND PRO v.4.21 (Pettersson Elektronik AB) to visually inspect all recorded sequences and remove those recordings that had (a) nonsearch‐phase calls, (b) calls not belonging to the targeted species, and (c) low signal‐to‐noise ratio. We distinguished search phase calls from approach‐phase and social calls by their duration, frequency, and pattern of change over time (Fenton, 2003; Schnitzler & Kalko, 2001). After the cleaning process, we selected the best call sequences with a minimum of five pulses each to be included in the final database.

## 3 RESULTS

We recorded 1,664 individual insectivorous bats and obtained a total of 1960 echolocation call sequences (each having at least five pulses). Hand release was the recording method with the highest number of calls with 1,236 (63%), followed by zip‐lining technique with 593 (30%; Table 2). *Pteronotus parnellii,* a species with Doppler‐shift compensation, was predominantly recorded inside a bag. Most molossids (particularly the genera *Molossus* and *Eumops*) were hand released at heights >5 m to prevent atypical FM calls produced when individuals are in the proximity of the ground and around obstacles (Table 2).

**TABLE 2:** Number of calls obtained per species with the different release methods used in Sonozotz

Species	Hand release	Zip‐lining	In bag	Flight cage	Flying from perch
Emballonuridae					
*Balantiopteryx io*	9				
*Balantiopteryx plicata*	40	2	1		
*Peropteryx kappleri*	5				
*Peropteryx macrotis*	22				
*Rhynchonycteris naso*	20	2			
*Saccopteryx bilineata*	30				
Molossidae					
*Eumops nanus*	1^a^				
*Eumops perotis*		1			
*Molossus alvarezi*	4^a^	1			
*Molossus molossus*	1			1	1
*Molossus rufus*	53^a^	1			
*Molossus sinaloae*	5				
*Nyctinomops aurispinosus*		3			
*Nyctinomops femorosaccus*					2
*Nyctinomops laticaudatus*	10				
*Nyctinomops macrotis*	2				
*Tadarida brasiliensis*	107	86		1	9
Mormoopidae					
*Mormoops megalophylla*	91	54	1		1
*Pteronotus davyi*	86	22	1		
*Pteronotus gymnonotus*	7	1			
*Pteronotus parnellii*	81	16	80	3	
*Pteronotus personatus*	38	11	1		
Natalidae					
*Natalus mexicanus*	34	6			
Noctilionidae					
*Noctilio leporinus*	8	1			
Phyllostomidae					
*Chrotopterus auritus*	7				
*Lampronycteris brachyotis*	1			1	
*Lonchorhina aurita*	1	1			
*Lophostoma brasiliense*	1			1	
*Macrotus californicus*	14	40	3		
*Macrotus waterhousii*	4				
*Micronycteris microtis*	5			2	
*Mimon cozumelae*	10	1			
*Phyllostomus discolor*	1				
*Trachops cirrhosus*	5				
*Vampyrum spectrum*				1	
Vespertilionidae					
*Antrozous pallidus*	28	52		1	
*Bauerus dubiaquercus*		1			
*Corynorhinus mexicanus*	8				
*Corynorhinus townsendii*	18	17			
*Eptesicus brasiliensis*	3				
*Eptesicus furinalis*	14	2			
*Eptesicus fuscus*	48	34	1	1	
*Idionycteris phyllotis*				1	
*Lasiurus blossevillii*	12	6		1	
*Lasiurus cinereus*	20	9			
*Lasiurus ega*	6	4			
*Lasiurus intermedius*	3				1
*Lasiurus xanthinus*	4	4			
*Myotis albescens*	5				
*Myotis auriculus*	6	3			
*Myotis californicus*	39	4			
*Myotis elegans*	2			6	
*Myotis fortidens*	5		1		
*Myotis keaysi*	33				
*Myotis melanorhinus*	11	10			
*Myotis nigricans*	40	16	1		
*Myotis occultus*				1	
*Myotis peninsularis*	12				
*Myotis thysanodes*	4	1			
*Myotis velifer*	143	136		6	
*Myotis vivesi*	19				
*Myotis volans*	11	11			
*Myotis yumanensis*	49	24			
*Parastrellus hesperus*	13	1			
*Perimyotis subflavus*	1				
*Rhogeessa aeneus*	11				
*Rhogeessa alleni*	2				
*Rhogeessa parvula*	11	5			
*Rhogeessa tumida*	10	4			

^a^ From an elevated place (e.g., building, bridge).

### 3.1 Taxonomic coverage

**FIGURE 3:**
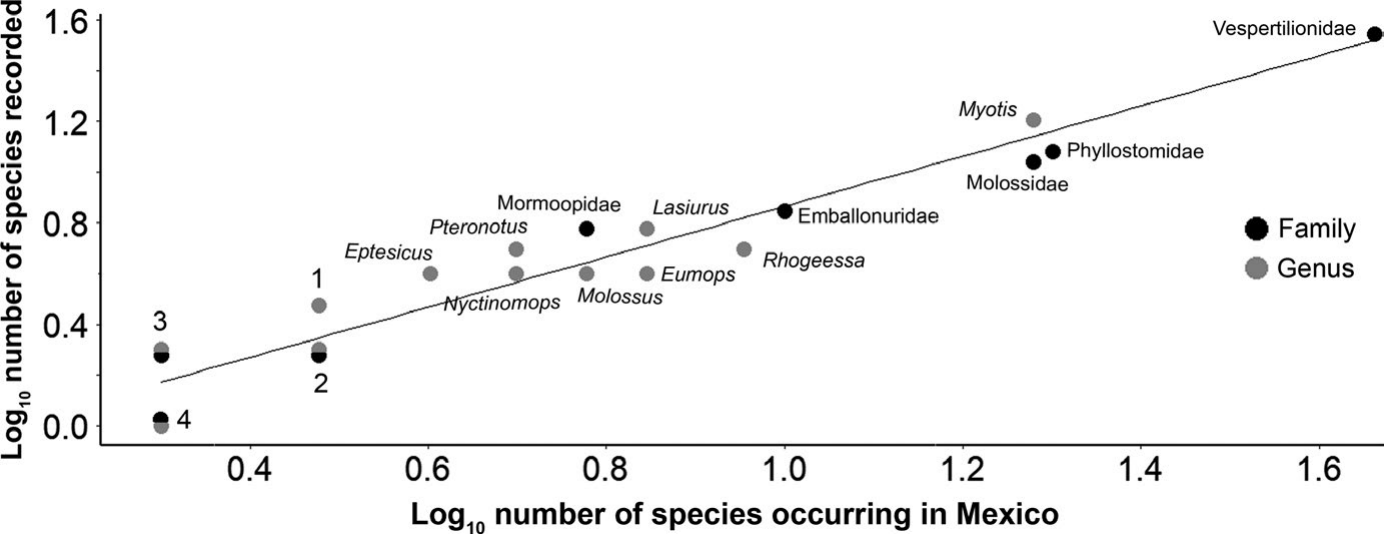
Taxonomic coverage represented by the number of species recorded in our project (per family and per genera) against the number of occurring species in Mexico. Families are shown in black and genera in gray

We recorded 69 insectivorous species (50% of total species and 64% of the predominantly insectivorous species found in Mexico) belonging to 33 genera (71% of genera from insectivorous bats that occur in Mexico) and 7 families (88% of the bat families occurring in Mexico). The species with the highest number of recorded individuals was *Myotis velifer* with 188 (11% of the total), followed by *Pteronotus parnellii* with 164 and *Tadarida brasiliensis* with 154 individuals (both 9%, Table 1). We obtained a single good quality echolocation call sequence for eleven species (Table 1). The family with most recordings was Vespertilionidae (*n* = 749), followed by Mormoopidae (*n* = 470). In contrast, the family Noctilionidae had the least number of individuals recorded (*n* = 9). Thyropteridae, represented in Mexico by a single species, *Thyroptera tricolor*, was the only family missing in the library. Mormoopidae and Vespertilionidae had the highest representation in our library in terms of species richness (Figure 3). On the other hand, the genera *Eumops, Molossus* (Molossidae), and *Rhogeessa* (Vespertilionidae) were underrepresented in our results (Figure 3).

### 3.2 Geographic and environmental coverage

**FIGURE 4:**
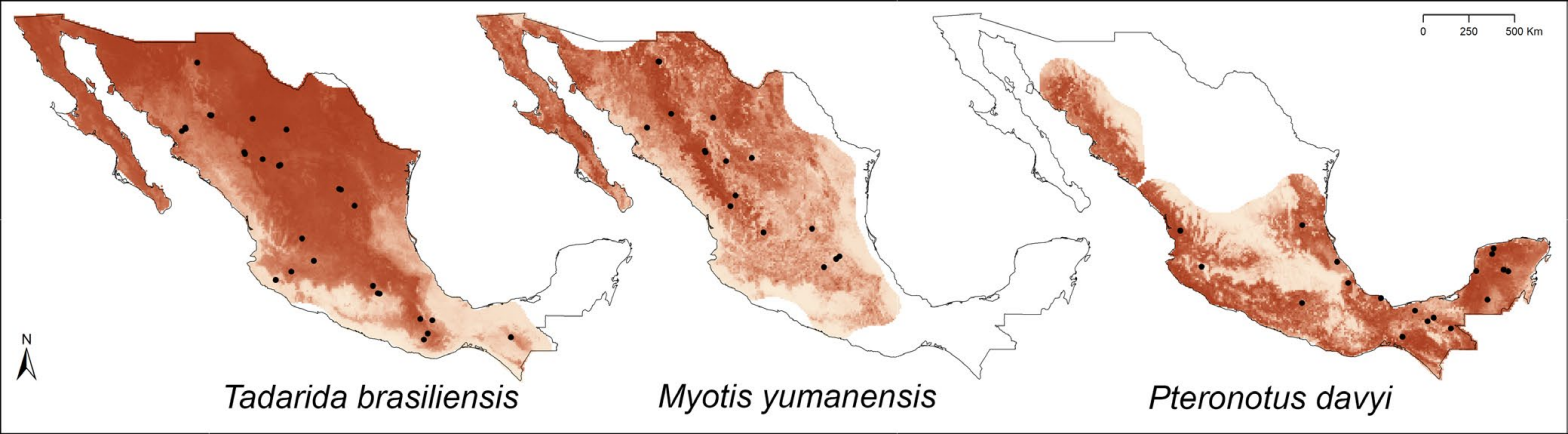
Examples of geographic coverage of the Sonozotz project. Maps show the distribution of sampling localities (black dots) for three bat species in relation to its geographic distribution (red area). At the left *Tadarida brasiliensis*, a member of the Molossidae family with a wide distribution all over Mexico; at the center *Myotis yumanensis*, a vespertilionid species with Nearctic affinity; and at the right *Pteronotus davyi*, a species with Neotropical affinity belonging to the Mormoopidae family

We obtained recordings from 109 sites and 185 localities covering the majority of Mexican ecoregions with the exception of the Great Plains (Figure 2). The tropical rainforest ecoregion had the highest number of sampling localities with 54, followed by the low tropical forest with 35 localities. The Californian Mediterranean ecoregion had only 4 sampling localities (Figure 2). Our example geographic coverage maps showed that *T. brasiliensis, M. yumanensis,* and *P. davyi* were adequately sampled along their distribution in the country (Figure 4). The “gaps” produced by lack of recordings observed along their distribution mainly correspond to areas where security reasons prevented sampling.

## 4 DISCUSSION

The Sonozotz project represents the most exhaustive effort to date to document and compile the diversity of bat echolocation calls for a megadiverse country. The high proportion of the Mexican bat fauna represented in the library (50% of total richness and 64% of insectivorous species) demonstrates that collaborative work among specialists can generate abundant data of remarkable scientific value that individual efforts cannot achieve. The large number of calls and sampling locations achieved exemplify the success of the project, representing properly the geographic and ecological variation of echolocation calls for Mexican insectivorous bats. The highly structured nature of the data and the standardized collecting methods carried out at each location were chosen to reduce the variation in bat calls due to methodological artifacts.

The Sonozotz library was designed to help address a range of ecological questions through the analysis of bat sound. Acoustic surveys have been successfully used to better understand the effects of anthropogenic activities and urbanization on bat communities, and to provide guidance on solutions for important current threats (i.e., wind development, white‐nose syndrome, environmental change) for bats worldwide (Berthinussen & Altringham, 2012; Bunkley, McClure, Kleist, Francis, & Barber, 2015; Estrada‐Villegas, Meyer, & Kalko, 2010; Jung & Kalko, 2011; Spoelstra et al., 2017). Sonozotz provides good quality calls with metadata that will help to determine the identity of taxonomic bat units with high precision. The amount of data collected is vast, providing a useful database for future analyses with different approaches (e.g., geographical variation, temporal call variation, and successional temporary assembly of a community) and for the use of mathematical algorithms for deeper analysis. Most importantly, uploading echolocation calls to the library is an ongoing process that will allow the increase in reference calls available for public use.

The first attempt to collate bat acoustic repositories dates from 1977 with the publication of Novick (1977), which summarizes the joint effort of a group of scientists to collect and describe echolocation calls for several bat species from different tropical countries. In the following years, the first specialized bat acoustic libraries appeared (e.g., Bat Conservation Trust Sound Library, Southeastern Australian Bat Call Library), but their use was limited because most of the material can only be listened to online or the material lacked environmental and methodological specifications. In other cases, the use of the description of bat echolocation calls was limited because the information was published in guides or identification keys that were available only in printed versions (e.g., Rainho, Amorim, Marques, & Rebelo, 2011; Reinhold, Law, Ford, & Pennay, 2001). These efforts were important to describe bat call diversity at local scales; however, they did not make the data freely available without methodological limitations. One of the biggest advances on bat acoustic repositories was Echobank (Collen, 2012), containing 53,488 calls in 3,531 call sequences, from 297 species belonging to 94 genera and 18 families. Nevertheless, tropical bats are still underrepresented in these databases (Walters et al., 2013). Several efforts have followed to assemble bat call libraries around the world, but many other countries and species remain undocumented.

Despite the popular use of classical parameters for bat acoustic phenotype description, several difficulties have arisen when implementing automated identifiers from this information source (Walters et al., 2013). Particularly, some groups exhibit high confusion rates, indicating a possible limit of identification cues that classical parameters can hold. Recently, the wider field of bioacoustic analysis has begun to incorporate classification algorithms that can use raw acoustic signals as input, such as deep neural networks (Strout et al., 2017), bypassing the use of predesigned features that could be suboptimal for classification purposes. Bat acoustic analysis is already turning to models that feed on raw acoustic inputs for detection tasks (Mac Aodha et al., 2018), and an analogous treatment of ultrasonic recordings for classification could improve automatic identification and help avoid the need of classical call parameters. The former trend suggests that access to proper reference material in a raw format will be a requisite for future bat acoustic analysis.

Improvements on the availability and accessibility of bat reference echolocation calls have allowed the establishment of community and citizen science monitoring programs at large spatial‐temporal scales, like iBats (Jones, Ryan, Flores, & Page, 2013) and the North American Bat Monitoring Program (NABat; Loeb et al., 2015). Having an adequate set of reference acoustic material is the first step to develop automatic identification tools that make feasible the analysis of large quantities of data (Walters et al., 2013). Sonozotz database will set the foundations to establish a national bat acoustic monitoring program and to develop an automatic call classifier in Mexico. Furthermore, Sonozotz can be an effective and useful dataset for researchers of both North America and South America, due to the country's peculiarity of sharing bat species with both regions.

### 4.1 Sonozotz coverage

Nine species of emballonurids occur in the country and six (66%) of them were represented in our dataset, being *Balantiopteryx plicata* the species with the highest number of call sequences (Table 1). The family Emballonuridae comprises species categorized as aerial insectivores that prefer to forage in open spaces and near water (Jung, Kalko, & von Helversen, 2007). Its foraging strategies make them hard to catch because species easily detect the mist net and usually forage high above the ground. Therefore, most of the individuals of this family were captured outside roosting sites. The echolocation calls obtained from members of this family had multiple harmonics with the highest energy placed in the second one, and with a characteristic signature of the family (Jakobsen, Kalko, & Surlykke, 2012).

We obtained recordings for over 50% of the molossid species occurring in Mexico. Although some genera were widely represented in the number of calls in our sampling (e.g., *Tadarida*), others (e.g., *Promops*, *Cynomops*, *Eumops*) were extremely difficult to capture because of their unknown roosting sites and their foraging strategy (i.e., aerial insectivory). In general, echolocation calls for molossids are typical of open space foragers with relatively low frequencies ranging from 7 to 35 kHz and long call durations up to 26 milliseconds (Jung et al., 2014; Kalko, Estrada‐Villegas, Schmidt, Wegmann, & Meyer, 2008). The structure of the echolocation calls of molossids has a strong phylogenetic component because it is shared in an orthological way between the different genera and species that make up the family (Jung et al., 2014).

The families Natalidae and Thyropteridae have only one representative in the country. For *Natalus mexicanus,* we obtained recordings from several geographic locations and environments. This genus produces multiharmonic frequencies with a marked energy accentuation in the second harmonic (Sanchez, Moreno, & Mora, 2017). In the case of *Thyroptera tricolor,* we could not obtain recordings of their calls due to the difficulty of their capture, which results from their occurrences at low densities, their highly ephemeral roosting's sites, and their highly specific habitat requirements (Chaverri & Gillan, 2015).

All the species of the family Mormoopidae were recorded and showed a high representation both in the geographical extent and in the number of individuals recorded. A typical mormoopid call consists of a constant frequency segment, followed by a modulated sweep descendent call, and finalizing with a quasi‐constant frequency with a short duration. It usually presents more than three strong harmonics with the highest intensity located on the second one (Ibañez, Guillen‐Servent, Juste, & Pérez‐Jordá, 1999).

The family Noctilionidae is represented in our dataset with two species within the country and is typically distributed in the riverine and lacustrine tropical areas of Mexico. We had a relatively high number of captured individuals of *Noctilio leporinus*, so we had a good representation of individuals recorded in the database. The calls of these fishing bats are typically long, with an initial constant frequency segment (54 kHz), followed by a frequency‐modulated (FM) component; this type of calls is usually emitted on the surface of the water for the search of prey at relatively short ranges (Hartley, 1989; Schnitzler, Kalko, Kaipf, & Grinnell, 1994). We could not collect any individual for *Noctilio albiventris.*

Most of the members of Phyllostomidae family emits echolocation calls (usually of low intensity) composed of multiharmonic components and constant modulated frequencies (Gessinger et al., 2019). The adaptability to understory orientation and the similarity among echolocation calls within this diverse group of bats makes almost impossible to detect particular signatures to identify species acoustically. Nevertheless, we recorded species of the Glyphonycterinae, Macrotinae, Micronycterinae, and Phyllostominae subfamilies because recent descriptions of some species show that they emit intense and distinct calls that could be included in monitoring programs (Brinkløv, Kalko, & Surlykke, 2010; Geipel, Jung, & Kalko, 2013; Gessinger et al., 2019; Weinbeer et al., 2013). This is particularly important as several members of these subfamilies are known to be sensitive to habitat disruption (Clarke, Rostant, & Racey, 2005; Fenton et al., 1992). Our database contains recordings for around 60% of the animalivore phyllostomids species occurring in Mexico. Echolocation calls for these bats are mainly composed by multiharmonic FM sweeps of short duration (Pio, Clarke, Mackie, & Racey, 2010).

Although the dry and cold winter months provided low or zero catches of vespertilionids in the center and north Mexico, we recorded 34 out of the 45 species (75%) of them occurring in the country. The Vespertilionidae family has the largest number of species and hold one of the finest and most sophisticated echolocation systems within Chiroptera because their ability to capture insects while flying and processing bouncing echoes at the same time (Ratcliffe, Elemans, Jakobsen, & Surlykke, 2013). Despite being a highly diverse family, their echolocation calls can be characterized by presenting broadband‐modulated frequencies, which are of relatively high intensity and short duration. The echolocation system of most vespertilionids is adapted to orientate and find food in cluttered environments (Fenton & Bell, 1979; Kingston, Jones, Akbar, & Kunz, 1999). The complexity of the echolocation calls, its high degree of specialization, and the diversification of sonogram topologies within the Vespertilionidae family, does not allow to characterize a standard call pattern for the family. However, the distinctive pulses do allow to study and identify some genera in fine detail and even some species. In Mexico, vespertilionids are not a dominant component of Neotropical bat assemblages, but they comprise a big part of the richness in the Nearctic bat communities.

### 4.2 Project constraints and future challenges

Although the Sonozotz database was targeted to cover high levels of intra‐ and interspecific variation under a standardized sampling protocol, it nonetheless has several limitations. The extreme environmental conditions in several sites in northern and central Mexico limited sampling during the winter period, as several species are known to hibernate (Ayala‐Berdón & Solís‐Cárdenas, 2017) or possibly migrate to other sites. The highest number of captures and recordings of insectivorous bats was carried out in the summer period (around 60%), but mainly in tropical rainforest habitats where bats are active year‐round.

Several “missing” species from our acoustic library are those that have an endemic distribution in Mexico (e.g., *Myotis findleyi, Myotis planiceps*) or those that occur in localities with low‐security conditions (e.g., *Lasionycteris noctivagans*, *Rhogeessa mira*). Other species have low detectability and capture rates, like many Glyphonycterinae, Micronycterinae, and Phyllostominae members, therefore requiring substantially more survey efforts (Meyer et al., 2011), which explains their absence in our library (e.g., *Macrophyllum* spp., *Tonatia* spp., *Glyphonycteris* spp.). Several species of molossids were not collected because their roosting sites are unknown or not accessible to sampling devices (e.g., *Promops centralis*, *Eumops underwoodi*) and also because they prefer to forage dozens, hundreds, or even thousands of meters from the ground, making them very hard to catch or record (Jung et al., 2014; McCracken et al., 2008). Specific field expeditions are required in the near future to obtain the echolocation calls of these particular and hard to catch species.

Sonozotz, a multi‐institutional project that involved the dedicated work of academics, students, NGO's and local people, is to our knowledge, the first largest effort to contain as much intra and interspecific call variation of insectivorous bats in a megadiverse country. Sonozotz recordings, in wav format, together with their associated metadata, will be freely available through the National Commission for the Knowledge and Use of Biodiversity (CONABIO). Once online, it will be possible to add new bat recordings, following a curatorial process to ensure the data quality and reliance of the new additions.

## CONFLICT OF INTEREST

The authors declare no conflict of interest.

## AUTHOR CONTRIBUTIONS

**Veronica Zamora‐Gutierrez:** Conceptualization (equal); Data curation (equal); Formal analysis (equal); Funding acquisition (equal); Investigation (equal); Methodology (equal); Project administration (equal); Resources (equal); Software (equal); Supervision (equal); Validation (equal); Visualization (equal); Writing‐original draft (equal); Writing‐review & editing (equal). **Jorge Ortega:** Conceptualization (equal); Data curation (equal); Formal analysis (equal); Funding acquisition (equal); Investigation (equal); Methodology (equal); Validation (equal); Writing‐original draft (equal); Writing‐review & editing. **Rafael Avila‐Flores:** Conceptualization (equal); Data curation (equal); Formal analysis (equal); Funding acquisition (equal); Investigation (equal); Methodology (equal); Writing‐original draft (equal). **Pedro Adrián Aguilar‐Rodríguez:** Data curation (equal); Investigation (equal); Writing‐review & editing (Supporting). **Martin Alarcón‐Montano:** Data curation (equal); Investigation (equal). **Luis Gerardo Avila‐Torresagatón:** Investigation (equal). **Jorge Ayala‐Berdón:** Investigation (equal). **Beatriz Bolívar‐Cimé:** Investigation (equal); Writing‐review & editing. **Miguel Briones‐Salas:** Conceptualization (equal); Data curation (equal); Formal analysis (equal); Funding acquisition (equal); Investigation (equal); Methodology (equal); Project administration (Lead); Supervision (equal). **Martha Chan‐Noh:** Investigation. **Manuel Chávez‐Cauich:** Investigation. **Cuauhtémoc Chávez:** Methodology. **Patricia Cortés‐Calva:** Conceptualization; Funding acquisition; Investigation. **Juan Cruzado:** Investigation. **Jesús Carlo Cuevas:** Investigation; Methodology. **Melina Del Real‐Monroy:** Investigation; Methodology; Writing‐review & editing. **Cynthia Elizalde‐Arellano:** Investigation; Writing‐review & editing‐Supporting. **Margarita García‐Luis:** Conceptualization; Funding acquisition; Investigation; Methodology; Project administration. **Rodrio García‐Morales:** Methodology; Writing‐review & editing. **José Antonio Guerrero:** Conceptualization; Data curation; Funding acquisition; Investigation; Methodology. **Aldo A. Guevara‐Carrizalez:** Investigation. **Edgar G. Gutiérrez:** Investigation. **Luis Arturo Hernández‐Mijangos:** Investigation. **Martha Pilar Ibarra‐López:** Investigation. **Luis Ignacio Íñiguez‐Dávalos:** Conceptualization; Data curation; Funding acquisition; Investigation; Methodology. **Rafael León‐Madrazo:** Investigation. **Celia López‐González:** Conceptualization; Data curation; Funding acquisition; Investigation; Methodology; Resources (equal); Writing‐review & editing. **M. Concepción López‐Tellez:** Investigation. **Juan Carlos López‐Vidal:** Investigation. **Santiago Martínez‐Balvanera:** Data curation; Formal analysis; Methodology; Software (equal). **Fernando Montiel‐Reyes:** Data curation; Formal analysis; Investigation; Methodology. **Rene Murrieta‐Galindo:** Investigation. **Carmen Lorena Orozco‐Lugo:** Conceptualization; Data curation; Funding acquisition; Investigation; Methodology. **Juan M. Pech‐Canché:** Conceptualization; Funding acquisition; Investigation; Methodology. **Lucio Pérez‐Pérez:** Investigation. **María Magdalena Ramírez‐Martínez:** Investigation. **Areli Rizo‐Aguilar:** Investigation. **Everardo Robredo‐Esquivelzeta:** Conceptualization; Data curation; Formal analysis; Methodology; Software (equal). **Alba Z. Rodas‐Martínez:** Data curation; Investigation; Writing‐review & editing. **Marcial Alejandro Rojo‐Cruz:** Investigation; Methodology. **Celia Isela Selem‐Salas:** Conceptualization; Data curation; Funding acquisition; Investigation; Methodology; Project administration. **Elena Uribe‐Bencomo:** Investigation. **Jorge A. Vargas‐Contreras:** Investigation; Writing‐review & editing. **Maria Cristina MacSwiney G.:** Conceptualization; Data curation; Formal analysis; Funding acquisition; Investigation; Methodology; Project administration; Resources‐equal; Supervision (equal); Validation (equal); Visualization (equal); Writing‐original draft; Writing‐review & editing.

## Data Availability

The metadata that support the findings of this study are available on Dryad public repository (www.dryad.org) through the following link: https://doi.org/10.5061/dryad.95x69p8g6. Additionally, metadata will be hosted by CONABIO through a public web app (selia.conabio.gob.mx) to be launched by mid 2020. This app will provide access to recordings and their metadata for consultation and download. It will also provide tools for visualization and annotation of acoustic data as well as access to other open ultrasonic and audible materials. Collected tissues were deposited in the Regional Collection of Durango (Mammalia), at CIDIIR‐Durango, and voucher specimens were deposited at the Mammalogy Collection, CIB at Centro de Investigaciones Biológicas del Noroeste (CIBNOR). Access to this material is upon request to the collection curators.

## References

[ece36245-bib-0001] Álvarez‐Castañeda, T. , Álvarez, T. , & González‐Ruiz, N. (2015). Keys for identifying Mexican mammals. Mexico: Centro de Investigaciones Biológicas del Noroeste, S.C., Escuela Nacional de Ciencias Biológicas, Universidad Autónoma Metropolitana, Unidad Iztapalapa.

[ece36245-bib-0002] Armitage, D. W. , & Ober, H. K. (2010). A comparison of supervised learning techniques in the classification of bat echolocation calls. Ecological Informatics, 5(6), 465–473. 10.1016/j.ecoinf.2010.08.001

[ece36245-bib-0003] Ayala‐Berdón, J. , & Solís‐Cárdenas, V. (2017). New record and site characterization of a hibernating colony of *Myotis velifer* in a mountain ecosystem of central Mexico. Therya, 8(2), 171–174. 10.12933/therya-17-469

[ece36245-bib-0004] Barclay, R. M. R. (1999). Bats are not birds: A cautionary note on using echolocation calls to identify bats: A comment. Journal of Mammalogy, 80(1), 290–296. 10.2307/1383229

[ece36245-bib-0005] Berthinussen, A. , & Altringham, J. (2012). Do bat gantries and underpasses help bats cross roads safely? PLoS ONE, 7(6), e38775 10.1371/journal.pone.0038775 22719941 PMC3374807

[ece36245-bib-0006] Blumstein, D. T. , Mennhill, D. J. , Clemins, P. , Girod, L. , Yao, K. , Patricelli, G. , … Kirschel, A. N. G. (2011). Acoustic monitoring in terrestrial environments using microphone arrays: Applications, technological considerations and prospectus. Journal of Applied Ecology, 48(3), 758–767. 10.1111/j.1365-2664.2011.01993x

[ece36245-bib-0007] Braun de Torrez, E. C. , Wallrichs, M. A. , Ober, H. K. , & McCleery, R. A. (2017). Mobile acoustic transects miss rare bat species: Implications of survey method and spatio‐temporal sampling for monitoring bats. Peer J, 5, e3940 10.7717/peerj.3940 29134138 PMC5682100

[ece36245-bib-0008] Brigham, R. M. , Kalko, E. K. V. , Jones, G. , Parsons, S. , & Limpens, H. J. G. A. (2004). Bat echolocation research: Tools, techniques and analysis. Austin, TX: Bat Conservation International.

[ece36245-bib-0009] Brinkløv, S. , Kalko, E. K. V. , & Surlykke, A. (2010). Dynamic adjustment of biosonar intensity to habitat clutter in the bat *Macrophyllum macrophyllum* (Phyllostomidae). Behavioral Ecology and Sociobiology, 64(4), 1867–1874. 10.1007/s00265-010-0998-9

[ece36245-bib-0010] Bunkley, J. P. , McClure, C. J. W. , Kleist, N. J. , Francis, C. D. , & Barber, J. R. (2015). Anthropogenic noise alters bat activity levels and echolocation calls. Global Ecology and Conservation, 3, 62–71. 10.1016/j.gecco.2014.11.002

[ece36245-bib-0011] Challenger, A. , & Soberón, J. (2008). Los ecosistemas terrestres In Sarukhán J., Koleff P., Carabias J., Soberón J., Dirzo R., Llorente‐Bousquets J., & de la Maza J. (comps.), Capital Natural de México. Vol. I: Conocimiento actual de la biodiversidad. México, D.F. México: Conabio.

[ece36245-bib-0012] Chaverri, G. , & Gillan, E. H. (2015). Repeatability in the contact calling system of Spix's disc‐winged bat (*Thyroptera tricolor*). Royal Society Open Science, 2(1), 140197 10.1098/rsos.140197 26064578 PMC4448792

[ece36245-bib-0013] Clarke, F. M. , Rostant, L. V. , & Racey, P. A. (2005). Life after logging: Postlogging recovery of a Neotropical bat community. Journal of Applied Ecology, 42(2), 409–420. 10.1111/j.1365-2664.2005.01024.x

[ece36245-bib-0014] Clement, M. J. , Murray, K. L. , Solick, D. I. , & Gruver, J. C. (2014). The effect of call libraries and acoustic filters on the identification of bat echolocation. Ecology and Evolution, 4(17), 3482–3493. 10.1002/ece3.1201 25535563 PMC4228621

[ece36245-bib-0015] Collen, A. L. (2012). The evolution of echolocation in bats: A comparative approach. Doctoral thesis, UCL (University College London).

[ece36245-bib-0016] Denzinger, A. , & Schnitzler, H.‐U. (2013). Bat guilds a concept to classify the highly diverse foraging and echolocation behaviors of microchiropteran bats. Frontiers in Physiology, 4, 164 10.3389/fphys.2013.00164 23840190 PMC3699716

[ece36245-bib-0017] Estrada‐Villegas, S. , Meyer, C. F. J. , & Kalko, E. K. V. (2010). Effects of tropical forest fragmentation on aerial insectivorous bats in a land‐bridge island system. Biological Conservation, 143(3), 597–608. 10.1016/j.biocon.2009.11.009

[ece36245-bib-0018] Fenton, M. B. (2003). Eavesdropping on the echolocation and social calls of bats. Mammal Review, 33(3–4), 193–204. 10.1046/j.1365-2907.2003.00019.x

[ece36245-bib-0019] Fenton, M. B. , Acharya, L. , Audet, D. , Hickey, M. B. C. , Merriman, C. , Obrist, M. K. , … Adkins, B. (1992). Phyllostomid bats (Chiroptera: Phyllostomidae) as indicators of habitat disruption in the Neotropics. Biotropica, 24(3), 440–446.

[ece36245-bib-0020] Fenton, M. B. , & Bell, G. P. (1979). Echolocation and feeding behaviour in four species of *Myotis* (Chiroptera). Canadian Journal of Zoology, 57(6), 1271–1277. 10.1139/z79-163

[ece36245-bib-0021] Fenton, M. B. , Portfors, C. V. , Rautenbach, I. L. , & Waterman, J. M. (1998). Compromises: Sound frequencies used in echolocation by aerial‐feeding bats. Canadian Journal of Zoology, 76(6), 1174–1182. 10.2307/2388615

[ece36245-bib-0022] Frick, W. (2013). Acoustic monitoring of bats, considerations of options for long‐term monitoring. Therya, 4(1), 69–78. 10.12933/therya-13-109

[ece36245-bib-0023] Geipel, I. , Jung, K. , & Kalko, E. K. V. (2013). Perception of silent and motionless prey on vegetation by echolocation in the gleaning bat *Micronycteris microtis* . Proceedings of the Royal Society B: Biological Series, 280, 20122830 10.1098/rspb.2012.2830 PMC3574334 23325775

[ece36245-bib-0024] Gessinger, G. , Gonzalez‐Terrazas, T. P. , Page, R. A. , Jung, K. , & Tschapka, M. (2019). Unusual echolocation behaviour of the common sword‐nosed bat *Lonchorhina aurita*: An adaptation to aerial insectivory in a phyllostomid bat? Royal Society Open Science, 6, 182165 10.1098/rsos.182165 31417705 PMC6689612

[ece36245-bib-0025] González‐Ruiz, N. , Ramírez‐Pulido, J. , & Arroyo‐Cabrales, J. (2011). A new species of Mastiff Bat (Chiroptera: Molossidae: Molossus) from Mexico. Mammalian Biology, 76(4), 461–469. 10.1016/j.mambio.2010.06.004

[ece36245-bib-0026] Hartley, D. J. (1989). The acoustic behavior of the fish‐catching bat, *Noctilio leporinus*, during prey capture. The Journal of the Acoustical Society of America, 86(8), 10.1121/1.398225

[ece36245-bib-0027] Hayes, J. (2000). Assumptions and practical considerations in the design and interpretation of echolocation‐monitoring studies. Acta Chiropterologica, 2, 225–236.

[ece36245-bib-0028] Hayes, J. , Ober, H. , & Sherwin, R. (2009). Survey and monitoring of bats In Kunz T., & Parsons S. (Eds.), Ecological and behavioral methods for the study of bats. Baltimore, MD: John Hopkins University Press.

[ece36245-bib-0029] Ibañez, C. , Guillen‐Servent, A. , Juste, J. , & Pérez‐Jordá, J. L. (1999). Echolocation Calls of *Pteronotus davyi* (Chiroptera: Mormoopidae) from Panama. Journal of Mammalogy, 80(3), 924–928. 10.2307/1383261

[ece36245-bib-0030] INEGI, CONABIO, and INE (2008). Retrieved from http://www.conabio.gob.mx/informacion/metadata/gis/ecort08gw.xml?_xsl=/db/metadata/xsl/fgdc_html.xsl&_indent=no

[ece36245-bib-0031] Jakobsen, L. , Kalko, E. K. V. , & Surlykke, A. (2012). Echolocation beam shape in emballonurid bats, *Saccopteryx bilineata* and *Cormura brevirostris* . Behavioral Ecology and Sociobiology, 66, 1493–1502. 10.1007/s00265-012-1404-6

[ece36245-bib-0032] Jones, G. , & Holderied, M. W. (2007). Bat echolocation calls: Adaptation and convergent evolution. Proceeding of the Royal Society B: Biological Series, 274(1612), 905–912. 10.1098/rspb.2006.0200 PMC1919403 17251105

[ece36245-bib-0033] Jones, G. , Jacobs, D. S. , Kunz, T. H. , Willig, M. R. , & Racey, P. A. (2009). Carpe noctem: The importance of bats as bioindicators. Endangered Species Research, 8(1), 93–115. 10.3354/esr00182

[ece36245-bib-0034] Jones, G. , & Teeling, E. (2006). The evolution of echolocation in bats. Trends in Ecology and Evolution, 21, 149–156. 10.1016/j.tree.2006.01.001 16701491

[ece36245-bib-0035] Jones, P. L. , Ryan, M. J. , Flores, V. , & Page, R. A. (2013). When to approach novel prey cues? Social learning strategies in frog‐eating bats. Proceedings of the Royal Society B: Biological Sciences, 280(1772), 20132330 10.1098/rspb.2013.2330 PMC3813345 24266035

[ece36245-bib-0036] Jung, K. G. , & Kalko, E. K. V. (2011). Adaptability and vulnerability of high flying Neotropical aerial insectivorous bats to urbanization. Diversity and Distributions, 17(2), 262–274. 10.1111/j.1472-4642.2010.00738.x

[ece36245-bib-0037] Jung, K. , Kalko, E. K. V. , & von Helversen, O. (2007). Echolocation calls in Central American emballonurid bats: Signal design and call frequency alternation. Journal of Zoology, 272, 125–137. 10.1111/j.1469-7998.2006.00250.x

[ece36245-bib-0038] Jung, K. , Molinari, J. , & Kalko, E. K. V. (2014). Driving factors for the evolution of species‐specific echolocation call design in New World free‐tailed bats (Molossidae). PLoS ONE, 9(1), e85279 10.1371/journal.pone.0085279 24454833 PMC3891751

[ece36245-bib-0039] Kalko, E. K. V. , Estrada‐Villegas, S. , Schmidt, M. , Wegmann, M. , & Meyer, C. F. (2008). Flying high‐assessing the use of aerosphere by bats. Integrative Comparative Biology, 48(1), 60–73. 10.1093/icb/icn030 21669773

[ece36245-bib-0040] Kalko, E. K. V. , Handley, C. O. , & Handley, D. (1996). Organization, diversity, and long‐term dynamics of a Neotropical bat community In Cody M. L., & Smallwood J. A. (Eds.), Long‐term studies in vertebrate communities (pp. 503–553). Los Angeles, CA: Academic Press.

[ece36245-bib-0041] Kalko, E. K. V. , & Schnitzler, H.‐U. (1993). Plasticity in echolocation signals of European Pipistrelle bats in search flight: Implications for habitat use and prey detection. Behavioral Ecology and Sociobiology, 33(6), 415–428.

[ece36245-bib-0042] Kingston, T. , Jones, G. , Akbar, Z. , & Kunz, T. H. (1999). Echolocation signal design in Kerivoulinae and Murininae (Chiroptera: Vespertilionidae) from Malaysia. Journal of Zoology, 249(3), 359–374. 10.1111/j.1469-7998.1999.tb00771.x

[ece36245-bib-0043] Liu, Z. , Cotton, J. A. , Shen, B. , Han, X. , Rossiter, S. J. , & Zhang, S. (2010). Convergent sequence evolution between echolocating bats and dolphins. Current Biology, 20(2), R53–R54. 10.1016/j.cub.2009.11.058 20129036

[ece36245-bib-0044] Loeb, S. C. , Rodhouse, T. J. , Ellison, L. E. , Lausen, C. L. , Reichard, J. D. , Irvine, K. M. , … Sauer, J. R. (2015). A plan for the North American bat monitoring program (NABat). General Technical Report, U.S. Department of Agriculture Forest Service, Southern Research Station, Asheville, NC.

[ece36245-bib-0045] López‐Wilchis, R. , Guevara‐Chumacero, L. , Pérez, N. , Juste, J. , Ibánez, C. , & Barriga‐Sosa, I. (2012). Taxonomic status assessment of the Mexican populations of funnel‐eared bats, genus *Natalus* (Chiroptera: Natalidae). Acta Chiropterologica, 14, 305–316. 10.3161/150811012X661639

[ece36245-bib-0046] Mac Aodha, O. , Gibb, R. , Barlow, K. E. , Browning, E. , Firman, M. , Freeman, R. , … Jones, K. E. (2018). Bat Detective—Deep learning tools for bat acoustic signal detection. PLoS Computational Biology, 14, 1–19. 10.1371/journal.pcbi.1005995 PMC5843167 29518076

[ece36245-bib-0047] McCracken, G. F. , Gillam, E. H. , Westbrook, J. K. , Lee, Y. , Jensen, M. L. , & Balsley, B. B. (2008). Brazilian free‐tailed bats (*Tadarida brasiliensis*: Molossidae, Chiroptera) at high altitude: Links to migratory insect populations. Integrative and Comparative Biology, 48(1), 107–118. 10.1093/icb/icn033 21669777

[ece36245-bib-0048] Medellín, R. A. , Arita, H. T. , & Sánchez, O. (2008). Identificación de los murciélagos de México. Clave de campo. Mexico, D.F: Instituto de Ecología, Universidad Nacional Autónoma de México.

[ece36245-bib-0049] Meyer, C. F. J. (2015). Methodological challenges in monitoring bat population‐ and assemblage‐level changes for anthropogenic impact assessment. Mammalian Biology, 80(3), 159–169. 10.1016/j.mambio.2014.11.002

[ece36245-bib-0050] Meyer, C. F. J. , Aguiar, L. M. S. , Aguirre, L. F. , Baumgarten, J. , Clarke, F. M. , Cosson, J. F. , … Kalko, E. K. V. (2011). Accounting for detectability improves estimates of species richness in tropical bat surveys. Journal of Applied Ecology, 48(3), 777–787. 10.1111/j.1365-2664.2011.01976.x

[ece36245-bib-0051] Mora, E. , & Torres, L. (2008). Echolocation in the Large Molossid Bats *Eumops glaucinus* and *Nyctinomops macrotis* . Zoological Science, 25(1), 6–13. 10.2108/zsj.25.6 18275247

[ece36245-bib-0052] Murray, K. L. , Britzke, E. R. , & Robbins, L. W. (2001). Variation in search‐phase calls of bats. Journal of Mammalogy, 82(3), 728–737. 10.1644/1545-1542(2001)082<0728:VISPCO>2.0.CO;2

[ece36245-bib-0053] Novick, A. (1977). Acoustic orientation In Wimsatt W. A. (Ed.), Biology of bats, Vol. III. New York, NY: Academic Press.

[ece36245-bib-0054] Obrist, M. K. (1995). Flexible bat echolocation: The influence of individual, habitat and conspecifics on sonar signal design. Behavioral Ecology and Sociobiology, 36(3), 207–219.

[ece36245-bib-0055] Park, K. J. (2015). Mitigating the impacts of agriculture on biodiversity: Bats and their potential role as bioindicators. Mammalian Biology, 80(39), 191–204. 10.1016/j.mambio.2014.10.004

[ece36245-bib-0056] Patriquin, K. J. , Hogberg, L. K. , Chruszcz, B. J. , & Barclay, R. M. R. (2003). The influence of habitat structure on the ability to detect ultrasound using bat detectors. Wildlife Society Bulletin, 31(2), 475–481.

[ece36245-bib-0057] Pio, D. , Clarke, F. M. , Mackie, I. , & Racey, P. A. (2010). Echolocation calls of the bats of Trinidad, West Indies: Is guild membership reflected in echolocation signal design? Acta Chiropterologica, 12(1), 217–229. 10.3161/150811010X504716

[ece36245-bib-0058] R Development Core Team (2018). R: A language and environment for statistical computing. Vienna, Austria: R Foundation for Statistical Computing.

[ece36245-bib-0059] Rainho, A. , Amorim, F. , Marques, J. T. , & Rebelo, H. (2011). Identification key to the vocalizations of bats of mainland Portugal. Lisbon, Portugal: Instituto da Conservacão da Natureza e das Florestas.

[ece36245-bib-0060] Ramírez‐Pulido, J. , González‐Ruiz, N. , Gardner, A. L. , & Arroyo‐Cabrales, J. (2014). List of recent land mammals from Mexico. Special Publications, Museum of Texas Tech University, 63, 1–69.

[ece36245-bib-0061] Ratcliffe, J. M. , Elemans, C. P. H. , Jakobsen, L. , & Surlykke, A. (2013). How the bat got its buzz. Biology Letters, 9, 20121031 10.1098/rsbl.2012.1031 23302868 PMC3639754

[ece36245-bib-0062] Reid, F. A. (2009). A field guide to the mammals of Central America & Southeast Mexico (2nd ed., p. 346). New York, NY: Oxford University Press.

[ece36245-bib-0063] Reinhold, L. , Law, B. , Ford, G. , & Pennay, M. (2001). Key to the Bat Calls of South‐east Queensland and North‐east New South Wales. Technical Report QNRM01001. Queensland Department of Natural Resources and Mines, Australia.

[ece36245-bib-0064] Russo, D. , & Jones, G. (2015). Bats as bioindicators: An introduction. Mammalian Biology, 80(3), 157–158. 10.1016/j.mambio.2015.03.005

[ece36245-bib-0065] Russo, D. , & Voigt, C. C. (2016). The use of automated identification of bat echolocation calls in acoustic monitoring: A cautionary note for a sound analysis. Ecological Indicators, 166, 598–602. 10.1016/j.ecolind.2016.02.036

[ece36245-bib-0066] Sanchez, L. , Moreno, C. R. , & Mora, E. C. (2017). Echolocation calls of *Natalus primus* (Chiroptera: Natalidae): Implications for conservation monitoring of this species. Cogent Biology, 3, 1355027 10.1080/23312025.2017.1355027

[ece36245-bib-0067] Schnitzler, H.‐U. , & Kalko, E. K. V. (2001). Echolocation by insect‐eating bats. BioScience, 51, 557–569. 10.1641/0006-3568(2001)051[0557:EBIEB]2.0.CO;2

[ece36245-bib-0068] Schnitzler, H.‐U. , Kalko, E. K. V. , Kaipf, I. , & Grinnell, A. D. (1994). Fishing and echolocation behavior of the greater bulldog bat, *Noctilio leporinus*, in the field. Behavioral Ecology and Sociobiology, 35(5), 327–345. 10.1007/BF00184422

[ece36245-bib-0069] Schnitzler, H.‐U. , Moss, C. F. , & Denzinger, A. (2003). From spatial orientation to food acquisition in echolocating bats. Trends in Ecology and Evolution, 18(8), 386–394. 10.1016/S0169-5347(03)00185-X

[ece36245-bib-0070] Siemers, B. M. , Beedholm, K. , Dietz, C. , Dietz, I. , & Ivanova, T. (2005). Is species identity, sex, age or individual quality conveyed by echolocation call frequency in European horseshoe bats? Acta Chiropterologica, 7(2), 259–274. 10.3161/1733-5329(2005)7[259:ISISAO]2.0.CO;2

[ece36245-bib-0071] Sikes, R. S. , & Animal Care and Use Committee of the American Society of Mammalogists (2016). Guidelines of the American Society of Mammalogists for the use of wild mammals in research and education. Journal of Mammalogy, 97(3), 663–688. 10.1093/jmammal/gyw078 29692469 PMC5909806

[ece36245-bib-0072] Smotherman, M. , & Guillén‐Servent, A. (2008). Doppler‐shift compensation behavior by Wagner’s mustached bat, *Pteronotus personatus* . Journal of the Acoustical Society of America, 123(6), 4331–4339. 10.1121/1.2912436 18537384 PMC2680666

[ece36245-bib-0073] Spoelstra, K. , van Grunsven, R. H. A. , Ramakers, J. J. C. , Ferguson, K. B. , Raap, T. , Donners, M. , … Visser, M. E. (2017). Data from: Response of bats to light with different spectra: Light‐shy and agile bat presence is affected by white and green, but not red light Dataverse Digital Repository, http://hdl.handle.net/10411/20867 10.1098/rspb.2017.0075 PMC5454258 28566484

[ece36245-bib-0074] Stahlschmidt, P. , & Brühl, C. A. (2012). Bats as bioindicators–the need of a standardized method for acoustic bat activity surveys. Methods in Ecology and Evolution, 3(3), 503–508. 10.1111/j.2041-210X.2012.00188.x

[ece36245-bib-0075] Strout, J. , Rogan, B. , Seyednezhad, S. M. M. , Smart, K. , Bush, M. , & Ribeiro, E. (2017). Anuran call classification with deep learning In *2017 IEEE International Conference on Acoustics, Speech and Signal Processing (ICASSP)* (pp. 2662–2665).

[ece36245-bib-0076] Szewczak, J. M. (2002). Analysis Techniques for identifying bat species In Mark Brigham R., Kalko E. K. V., Jones G., Parsons S., & Limpens H. J. G. A. (Eds.), Bat echolocation research: tools, techniques and analysis (pp. 121–127). Austin, TX: Bat Conservation International.

[ece36245-bib-0077] Thomas, D. , Bell, G. , & Fenton, M. (1987). Variation in echolocation call frequencies recorded from North American vespertilionid bats: A cautionary note. Journal of Mammalogy, 68(4), 842–847. 10.2307/1381562

[ece36245-bib-0078] Ulanovsky, N. , Fenton, M. B. , Tsoar, A. , & Korine, C. (2004). Dynamics of jamming avoidance in echolocating bats. Proceeding of the Royal Society B: Biological Series, 271(1547), 1467–1475. 10.1098/rspb.2004.2750 PMC1691745 15306318

[ece36245-bib-0079] Walters, C. L. , Collen, A. , Lucas, T. , Mroz, K. , Sayer, C. A. , & Jones, K. (2013). Challenges of using bioacoustics to globally monitor bats In Adams R. A., & Pedersen S. C. (Eds.), Bat evolution, ecology and conservation (pp. 479–499). New York, NY: Springer.

[ece36245-bib-0080] Weinbeer, M. , Kalko, E. K. V. , & Jung, K. (2013). Behavioral flexibility of the trawling long‐legged bat, *Macrophyllum macrophyllum* (Phyllostomidae). Frontiers in Physiology, 4, 1–11. 10.3389/fphys.2013.00342 24324442 PMC3838978

[ece36245-bib-0081] Zamora‐Gutierrez, V. , Lopez‐Gonzalez, C. , MacSwiney Gonzalez, M. C. , Fenton, B. , Jones, G. , Kalko, E. K. V. , … Jones, K. E. (2016). Acoustic identification of Mexican bats based on taxonomic and ecological constraints on call design. Methods in Ecology and Evolution, 7(9), 1082–1091. 10.1111/2041-210X.12556

[ece36245-bib-0082] Zamora‐Gutierrez, V. , Pearson, R. G. , Green, R. E. , & Jones, K. E. (2018). Forecasting the combined effects of climate and land use change on Mexican bats. Diversity and Distributions, 24(3), 363–374. 10.1111/ddi.12686

